# Supplementary material for: Role and mechanism of NCAPD3 in promoting malignant behaviors in gastric cancer
Source: Front Pharmacol. 2024 Apr 22;15:1341039. doi: 10.3389/fphar.2024.1341039 (PMC11070777; doi:10.3389/fphar.2024.1341039)
Supplement: Supplementary file 11 [file DataSheet2.ZIP › GSEA/Oncogenic signature/my_analysis.Gsea.1599461962066/ERBB2_UP.V1_UP.html]

Details for gene set ERBB2\_UP.V1\_UP[GSEA]

|  || Dataset | filtered\_dataset.sample\_info.cls#WT\_versus\_NCAPD3\_MUT |
| Phenotype | sample\_info.cls#WT\_versus\_NCAPD3\_MUT |
| Upregulated in class | NCAPD3\_MUT |
| GeneSet | ERBB2\_UP.V1\_UP |
| Enrichment Score (ES) | -0.39476985 |
| Normalized Enrichment Score (NES) | -2.079084 |
| Nominal p-value | 0.0024570024 |
| FDR q-value | 0.019468369 |
| FWER p-Value | 0.08 |
Table: GSEA Results Summary

  

Fig 1: Enrichment plot: ERBB2\_UP.V1\_UP      
 Profile of the Running ES Score & Positions of GeneSet Members on the Rank Ordered List

  

| SYMBOL | TITLE | RANK IN GENE LIST | RANK METRIC SCORE | RUNNING ES | CORE ENRICHMENT || 1 | 79789 | CLMN | 160 | 0.717 | -0.0674 | No |
| 2 | 64856 | VWA1 | 259 | 0.623 | -0.0962 | No |
| 3 | 1601 | DAB2 | 327 | 0.577 | -0.1058 | No |
| 4 | 81031 | SLC2A10 | 539 | 0.453 | -0.2279 | No |
| 5 | 6919 | TCEA2 | 712 | 0.368 | -0.3276 | No |
| 6 | 25825 | BACE2 | 756 | 0.344 | -0.3354 | No |
| 7 | 6558 | SLC12A2 | 839 | 0.276 | -0.3761 | Yes |
| 8 | 6303 | SAT1 | 850 | -0.233 | -0.3676 | Yes |
| 9 | 360 | AQP3 | 857 | -0.263 | -0.3542 | Yes |
| 10 | 55450 | CAMK2N1 | 861 | -0.269 | -0.3383 | Yes |
| 11 | 9582 | APOBEC3B | 868 | -0.278 | -0.3238 | Yes |
| 12 | 3913 | LAMB2 | 919 | -0.348 | -0.3365 | Yes |
| 13 | 3691 | ITGB4 | 926 | -0.352 | -0.3171 | Yes |
| 14 | 4680 | CEACAM6 | 955 | -0.374 | -0.3121 | Yes |
| 15 | 6286 | S100P | 1011 | -0.412 | -0.3241 | Yes |
| 16 | 11138 | TBC1D8 | 1026 | -0.424 | -0.3056 | Yes |
| 17 | 1543 | CYP1A1 | 1058 | -0.451 | -0.2976 | Yes |
| 18 | 5097 | PCDH1 | 1132 | -0.502 | -0.3166 | Yes |
| 19 | 604 | BCL6 | 1136 | -0.504 | -0.2847 | Yes |
| 20 | 831 | CAST | 1160 | -0.524 | -0.2660 | Yes |
| 21 | 3887 | KRT81 | 1168 | -0.536 | -0.2349 | Yes |
| 22 | 2119 | ETV5 | 1176 | -0.545 | -0.2032 | Yes |
| 23 | 6310 | ATXN1 | 1177 | -0.547 | -0.1662 | Yes |
| 24 | 665 | BNIP3L | 1185 | -0.556 | -0.1338 | Yes |
| 25 | 23508 | TTC9 | 1271 | -0.660 | -0.1508 | Yes |
| 26 | 80328 | ULBP2 | 1277 | -0.664 | -0.1095 | Yes |
| 27 | 10397 | NDRG1 | 1380 | -0.925 | -0.1210 | Yes |
| 28 | 64764 | CREB3L2 | 1382 | -0.930 | -0.0589 | Yes |
| 29 | 1475 | CSTA | 1400 | -1.162 | 0.0072 | Yes |
Table: GSEA details [plain text format]

  

Fig 2: ERBB2\_UP.V1\_UP      
 Blue-Pink O' Gram in the Space of the Analyzed GeneSet

  

Fig 3: ERBB2\_UP.V1\_UP: Random ES distribution      
 Gene set null distribution of ES for **ERBB2\_UP.V1\_UP**
